# Rural Australian community pharmacists' views on complementary and alternative medicine: a pilot study

**DOI:** 10.1186/1472-6882-11-103

**Published:** 2011-10-28

**Authors:** Nicole J Bushett, Virginia A Dickson-Swift, Jon A Willis, Pene Wood

**Affiliations:** 1 School of Pharmacy and Applied Science, Faculty of Science, Technology and Engineering. La Trobe University, Bendigo, Victoria, Australia; 2 Department of Health and Environment, Faculty of Health Science, La Trobe Rural Health School, Bendigo, Victoria, Australia

## Abstract

**Background:**

Complementary and alternative medicines (CAMs) are being used increasingly across the world. In Australia, community pharmacists are a major supplier of these products but knowledge of the products and interactions with other medicines is poor. Information regarding the use of CAMs by metropolitan pharmacists has been documented by the National Prescribing Service (NPS) in Australia but the views of rural/regional community pharmacists have not been explored. The aim of this pilot study was to explore the knowledge, attitudes and information seeking of a cohort of rural community pharmacists towards CAMs and to compare the findings to the larger NPS study.

**Methods:**

A cross sectional self-administered postal questionnaire was mailed to all community pharmacists in one rural/regional area of Australia. Using a range of scales, data was collected regarding attitudes, knowledge, information seeking behaviour and demographics.

**Results:**

Eighty eligible questionnaires were returned. Most pharmacists reported knowing that they should regularly ask consumers if they are using CAMs but many lacked the confidence to do so. Pharmacists surveyed for this study were more knowledgeable in regards to side effects and interactions of CAMs than those in the NPS survey. Over three quarters of pharmacists surveyed reported sourcing CAM information at least several times a month. The most frequently sought information was drug interactions, dose, contraindications and adverse effects. A variety of resources were used to source information, the most popular source was the internet but the most useful resource was CAM text books.

**Conclusions:**

Pharmacists have varied opinions on the use of CAMs and many lack awareness of or access to good quality CAMs information. Therefore, there is a need to provide pharmacists with opportunities for further education. The data is valuable in assisting interested stakeholders with the development of initiatives to address the gaps in attitudes, knowledge and to improve effectiveness of information seeking behaviour.

## Background

Complementary and alternative medicines (CAMs) are defined as 'herbal medicines, vitamin and mineral supplements, other nutritional supplements, traditional medicines such as Ayurvedic medicines and traditional Chinese medicines (TCM), homeopathic medicines and aromatherapy oils where they make a therapeutic claim' [1]. CAMs lie outside mainstream medical care within Australia, however, the popularity of CAMs in Australia is rising with up to 70% of the population reporting using some type of CAM [2-7]. Most users of CAMs believe that the role of the pharmacist should include the provision of safety information, routine checks for drug interactions and recommendations of effective CAMs [7].

Research investigating pharmacists' attitudes to CAMs has been conducted within Australia but not specifically in rural regions [8]. Community pharmacists are one of the main suppliers of CAMs in Australia responsible for around 40% of total sales [3,5]. Overall, pharmacists' attitudes to statements about CAMs indicate they are cautious and concerned about issues such as safety, efficacy and regulation of the medicines [9]. In addition, pharmacists believe there is a lack of quality information and limited access to evidence based information [10]. Therefore, they are not confident in discussing these medicines with their patients [4]. An Australian study within New South Wales found that pharmacists' attitudes towards the broader concept of CAMs was positive [11]. Three-quarters of their respondents believed that CAMs are a useful supplement to conventional medicine. Furthermore, a survey of pharmacists in the United States of America found 80% of respondents agreed that 'some herbal or natural products work and would recommend them to patients' [12].

There is evidence to suggest that pharmacists' knowledge is inadequate when counselling patients on CAMs [13]. Studies in Australia, the United Kingdom, the United States of America and Singapore have all acknowledged that pharmacists rate their knowledge and skills to counsel patients on CAMs as inadequate [6,14-17]. There are many compounding factors for this lack of knowledge. Firstly, it is not mandatory to implement CAM teaching into pharmacy courses and the extent to which CAM is covered varies [18,19]. It has been found that educational exposure is directly correlated with the perceived usefulness of CAMs [20]. Therefore, students had a balanced view on the systems of healthcare outside of conventional medicine [21]. Despite varied teaching, there is a strong interest in learning more about CAMs at a tertiary level from both pharmacy and medical students [20,22,23]. Secondly, Australian pharmacists believe there is a lack of accurate and easily accessible information, including good patient resources [13]. Pharmacists sought integrated non-biased and evidence-based information, especially as many community pharmacists do not have access to CAM information resources [11,13]. It is difficult for pharmacists to counsel patients on CAMs when the medical fields' knowledge of drug interactions is lacking [24]. It is these omissions that have been found to negatively impact on community pharmacists' knowledge of CAMs [21].

A study examining pharmacists' attitudes, knowledge and information seeking behaviour towards CAMs was undertaken in 2008 by the National Prescribing Service (NPS)[8]. The National Prescribing Service Ltd (NPS) is an independent, non-profit organisation funded by the Australian Government Department of Health and Ageing which aims to improve quality use of medicines in Australia by giving people information, skills and knowledge so they can choose if, when and how to use medicines to attain better health and wellbeing Almost all of the community pharmacists surveyed by the NPS had recommended some kind of CAM in the last 12 months; however, they were not often involved in CAM sales. The pharmacists' attitudes were in favour of CAMs needing more scientific testing before using them in conventional medicine. In addition, less than half of community pharmacists were confident discussing CAMs with patients. In the NPS study three CAMs were used to assess knowledge, one which is commonly used (glucosamine) and two where there is evidence of potential adverse effects and interactions with conventional medicines (black cohosh and gingko biloba). The results of the knowledge test showed that based on self-rated knowledge, two-thirds of pharmacists believed they had a good working knowledge of glucosamine. One-third stated they had minimal or no knowledge about black cohosh and 15% reported having minimal or no knowledge about gingko biloba. Likewise, actual knowledge of key side effects and potential drug interactions for these medications varied greatly. On the subject of information seeking behaviour by pharmacists, the general consensus was that they tried to gather CAMs information from a variety of different sources, however a number of these resources were considered relatively useless.

Whilst the findings from the NPS study provide some information and understanding about CAM use and knowledge the study population was predominately limited to metropolitan pharmacists, which does not provide an overview of rural pharmacists. Therefore, the current study focused solely on a rural population for the following reasons. Firstly, community pharmacists play an important role in maintaining rural health because access to primary health care is often limited in rural areas [25-28]. Studies in Australia and North America have reported higher consumption of CAM in rural populations relative to their urban counterparts [29,30]. Furthermore, rural populations in Australia are commonly of lower socio-economic and health status than those living in urban areas, despite constituting approximately 32% of the Australian population [31,32]. Secondly, pharmacists working within rural areas often work independent of other health professionals and hence are less able to rely on other health professionals and may have limited access to resources [25-28]. It is important to ascertain their level of knowledge and their information seeking behaviour. That way, if deficits in knowledge are identified then resources may be developed in order to improve knowledge and additionally, governing pharmacy bodies can provide support [1]. It is important to determine the current state of knowledge and attitudes of rural pharmacists towards CAMs, primarily because they are isolated and require greater access to resources [33]. It is ultimately the patient who suffers if the pharmacist is unable to provide the most appropriate and relevant information.

The aims of this study are to determine rural community pharmacists':

1. Attitudes to CAMs

2. Knowledge of CAMs

3. Information seeking behaviour in relation to CAMs

The study also seeks to compare the findings with those derived from the study conducted by the NPS in 2008, which predominately assessed metropolitan community pharmacists.

## Methods

In order to ascertain the views of rural community pharmacists a pilot study was undertaken using a cross-sectional questionnaire as part of a quantitative research design [34]. The aim of the pilot study was to test the tool used by the NPS for use in this population and to make some comparisons between the studies. The data was collected from June to September 2010 using a cross-sectional self-administered postal questionnaire distributed to all community pharmacists within the Loddon Mallee region of Victoria registered with the Australian Pharmacy Board. This region was selected as it is the largest rural region in terms of geographic area within Victoria [35]. This type of study was selected as there has been little research conducted within Australia on rural community pharmacists' and CAMs. and to assess if the tool was useful for this cohort [36].

### Participants

The target population was all community pharmacists within the Loddon Mallee Region. The population was selected from a register of all pharmacists within the region, which totalled 252 people. For the purposes of this study the pharmacists' were required to be working primarily in a community pharmacy within the identified region. Primarily refers to the area of practice in which they worked most often, with a cut-off ratio of 2:1 in terms of hours they work. For example, pharmacists working in community pharmacy for 20 hours per week and hospital pharmacy for 10 hours per week have a ratio of 2:1 and would be classified as community pharmacists. However, working 15 hours per week in a community pharmacy and 10 hours per week in a hospital pharmacy would be a ratio of 1.5:1 and therefore they were excluded from the study

### Data collection

Questionnaires are the most widely used approach by pharmacy practice researchers, accounting for a higher proportion of published papers than any other [37,38]. Data was collected via an anonymous, self-administered postal questionnaire that was adapted from NPS study [39] (Questionnaire provided as Additional file). All community pharmacists within the region were sent a pack that included a questionnaire, reply paid envelope and participant information sheet. Dillmann's method of follow up was utilised in an attempt in order to improve response rates [40,41]. Potential respondents were contacted a fortnight after the original questionnaire was mailed out and any non-responders were sent a final questionnaire two weeks later.

The questionnaire consisted of 26 core questions which collected demographic and practice related information. It also included a range of scales to measure knowledge, experience, attitudes and information seeking behaviour with regard to CAMs. The response options varied depending on the types of question asked and included multiple choice, open-ended questions and Likert- style scaled responses. The study had ethical approval from La Trobe University, FHEC 10/R46.

### Data Analysis

The questionnaire responses were analysed using the Statistical Package for Social Sciences (SPSS) for Windows version 19 and Microsoft Office Excel 2007. Frequencies and percentages of responses were generated for each answer in the questionnaire. If responses were continuous and numerical, descriptive statistics were generated (mean, standard deviation, median, minimum and maximum). Univariate and bivariate analyses were conducted to explore CAM recommendations, attitudes, knowledge and information seeking behaviours. Analyses of associations between categorical responses using cross tabulation to compare knowledge of side effects and drug interactions with recommenders of CAMs were tested using chi square [42].

## Results

### Respondents

In total, 252 questionnaires were mailed, 110 were returned (response rate of 43.6%) Thirty of those were deemed ineligible as the pharmacists identified that they were either working part-time or working predominately in the hospital sector which gave an overall response rate of 31.7%) The demographic and practice information of the respondents are provided in Table 1.

**Table 1:** Demographic and practice characteristics of respondents

Demographic and practice characteristics		Community pharmacists (n = 80) %
Gender	Male	35.0

	Female	65.0

Age (years)	< 35	39.7

	35-44	20.5

	45-54	25.6

	55-64	10.3

	≥ 65	3.8

Trained in Australia		90%

Postgraduate CAM qualifications		10%

Survey respondents reported practising as pharmacists for 16.8 years ± 13.2 years and the majority worked full time (33 hours per week ± 13 hours per week with distribution negatively skewed; median 38 hours per week). Nineteen percent worked as consultants or accredited pharmacists for an average of 9.6 hours per week (SD: 13.2) compared to 14% in the NPS study. Note that accredited pharmacists are those that are registered with the Pharmacy Board of Australia. Forty-five percent of survey respondents, considered that they practised integrative care, defined as 'a holistic approach to health care that integrates conventional medical care with complementary therapies'.

### Attitudes towards Complementary and alternative medicine

Pharmacists were asked about their attitudes towards using CAMs. In the previous 12 months, 77.5% of pharmacists reported personally using CAMs. Table 2. provides a summary of pharmacists' attitudes towards various statements on CAMs. Further

**Table 2:** Attitudes of pharmacists towards complementary and alternative medicine

Statements		Community pharmacists (n = 80)	
	
	Strongly agree or agree %	Strongly disagree or disagree %	Neither agree nor disagree %
CAMS need more scientific testing before being used in conventional medicine	76.3	6.00	15.0

CAMS have a more holistic approach to health than conventional medicines	40.0	28.8	30.0

Most CAMS are safe and have very few side effects	26.3	53.8	18.8

The results from CAMs are mainly due to a placebo effect	12.5	47.5	35.0

CAMs can offer consumers benefits that conventional medicine cannot	42.6	21.3	35.0

Pharmacists should regularly ask consumers if they are using CAMs	93.8	0.00	5.00

I am confident discussing CAMs therapy with consumers	52.6	31.3	15.0

• Not all columns add up to 100% which indicates a lack of response to that question

analysis of the relationship between years of experience as a pharmacist and confidence in discussing CAMs with consumers highlighted that those respondents with 9 years or less of experience felt confident in discussing CAMs with patients. Interestingly, those who have 25-29 years of experience as a pharmacist were as confident but many of them also reported completing post graduate CAM qualifications. The least confident age group were those who had 10-14 years of experience as a pharmacist.

Recommendation rates of CAMs in the previous 12 months were high with 95% of pharmacists reporting recommending CAMs to a consumer. These results show that whilst a pharmacist may or may not be confident or adequately equipped with knowledge of CAMs, they are still recommending them. In other results, 65% of community pharmacists indicated they were involved in less than half of the CAMs sales in their community pharmacy. When they were involved in sales, most community pharmacists (88.2%) usually recommended the generic name of the CAM only, and for the minority who recommended specific brands, brand familiarity was the main influence on their decision.

All of the community pharmacists had discussed CAMs with patients. However, most of the time it was patient initiated either in the context where a patient requested CAMs (95%) or the patient telling the pharmacist they are taking a CAM (76.25%). Another situation suggested by respondents was when a pharmacy assistant refers to the pharmacist during the OTC sale (2.5%).

### Knowledge

The results of a knowledge test to be completed without using references aimed to ascertain what pharmacists knew about CAMs. The questions assessed self-rated knowledge, side effects and drug interactions of three CAMs, one which is commonly used (glucosamine) and two (black cohosh and gingko biloba) where there is evidence of potential adverse effects and interactions with conventional medicines.

The results of the self-rated knowledge questions show that over two thirds of pharmacists believe they have a good working knowledge of glucosamine, and one-third knows a little about its uses, side effects and drug interactions. However, the situation is reversed for black cohosh and gingko biloba where the majority of pharmacists indicate they know only minimal details about these CAMs.

Pharmacists' knowledge of some potentially serious side effects are shown in Table 3. 40.3% of pharmacists were aware of the potential for black cohosh to cause liver toxicity as an adverse effect. 56.2% of pharmacists knew that gingko biloba can cause bleeding disorders, however other potential side effects of the three CAMs were known by less than half of the pharmacist respondents. For all CAM side effects, those pharmacists who recommended the specific CAM were more likely to know of the side effect than non recommenders. However, this was only statistically significant for pharmacists who recommended gingko biloba.

**Table 3:** Community pharmacists' knowledge about specific side effects for three CAMs

CAM side effect		Community pharmacists' knowledge of the side effect	
	**All respondents %** **(n = 80)**	**Recommenders** **%**	**Non- Recommenders** **%**

Black cohosh - liver problems	40.3	42.3	39.1

Gingko biloba - bleeding disorders	56.2	63.9	48.6

Gingko biloba - seizures	15.1	19.4	10.8

Gingko biloba - dizziness/headache*	49.3	61.0	37.8

Black cohosh - dizziness/headache	27.8	38.5	21.7

NB: **N.B**.*Difference between non recommenders and recommenders p < 0.01 with a chi square value of 19.9, 4df.

Between 25% and 70% of pharmacists indicated that they were unsure of the possible side effects of these 3 CAMs. All pharmacists believed there are potential side effects of black cohosh and only 1.3% believed there were no side effects associated with using gingko biloba. However, a significant proportion of pharmacists were unsure of the potential side effects of glucosamine (68.4%).

Pharmacists' knowledge of some potential interactions between the selected CAMs and some conventional medicines was also assessed. Over 40% of pharmacists correctly identified the interaction between glucosamine and warfarin and 76% were aware of the interaction between gingko biloba and warfarin. Pharmacists who recommended the specific CAMs in the last 12 months were more likely to correctly answer knowledge of drug interactions.

A significant proportion, between one-third and one-half, of community pharmacists were unsure of the possible drug interactions of these three CAMs. All respondents correctly stated there are potential interactions with black cohosh, however, 48% were still unsure of the potential interactions. Close to one-fifth of participants incorrectly indicated there were no potential for interactions with glucosamine.

### Information seeking behaviour

Almost all pharmacists (97.5%) had sought information about CAMs in the last 12 months. In addition, over three-quarters of pharmacists sought CAM information several times a month or more frequently. The most frequently sought after information related to the safety of CAMs, which included drug interactions (95%), contraindications (76%) and adverse effects (75%).

Pharmacists used a wide range of information resources for finding out information on CAMs. The most popular were internet searches, MIMS/APP Guide, specific websites and APF. However, there was disparity between usage and perceived usefulness of these CAM resources. The resources ranked highest in terms of usefulness were CAM text books, internet searches and specific websites. A total of 93% of pharmacists had used the Internet in the last 12 months to seek information on CAMs.

Pharmacists were asked to choose, from a list, which CAMs they would like to have good quality information on and to indicate any other which was not listed. The top five were coenzyme Q10 (81%), glucosamine (70%), fish oil (68%), gingko biloba (68%) and echinacea (60%). Others included saw palmetto and probiotics at 6.3%.

## Discussion

Although other studies have investigated Australian pharmacists' experiences with CAMs, this study aimed to provide a snapshot of the knowledge, attitudes and information seeking behaviour of a cohort of rural community pharmacists to CAMs [8]. The results provide a clear indication of current information gaps that need to be addressed and it is an important addition to the body of knowledge of CAMs in relation to community pharmacists.

The socio-demographic characteristics of participating community pharmacists as presented in Table 1 are comparable to national pharmacy workforce data. Sixty five percent of the respondents were female, 39.7% were under 35 years of age with more than half being less that 44 years of age (60.4%). These figures reflect the national data reported by the Pharmacy Guild of Australia in their latest workforce planning study [43].

In the current study the age of respondents ranged from 24 to 74 years (mean age was 40.6 years; SD: 12.9 years). Overall, compared to the NPS study, pharmacists in the current study were on average younger and therefore had less professional experience. It was also interesting to note that 19% also worked as consultants or accredited pharmacists for an average of 9.6 hours per week (SD: 13.2) compared to 14% in the NPS study. Only 45% of respondents, compared to approximately half of NPS respondents, considered that they practised integrative care, defined as 'a holistic approach to health care that integrates conventional medical care with complementary therapies'. Finally, the NPS study had only 2.7% of respondents who have undertaken postgraduate CAM qualifications compared to 10% in the current study.

### Attitudes

The findings of this study are similar to many other studies that have found that many pharmacists lack confidence in discussing CAMs and have concerns with safety related to poor knowledge [9-12,15,17]. Almost all pharmacists in the current study agreed that they should regularly ask consumers if they are using CAMs. However, only half felt confident in discussing CAM therapy with consumers. An Australian survey conducted in 2004 found that less than 15% of community pharmacists reported feeling very confident in answering enquiries about the safety, interactions, adverse effects and benefits of CAMs [13]. In the current study there were a significant proportion of pharmacists who felt CAMs required further scientific testing before being used in conventional medicine, as was also the case in the NPS study. In addition, many pharmacists were concerned about the safety of CAM use as 57% disagreed that most CAMs are safe and have very few side effects.

In comparison to the NPS study, participants in the current study had a greater percentage of pharmacists strongly agreeing or agreeing that most CAMs are safe (26.3% versus 17.4% in the NPS study). Between the two studies, the current study has a lesser percentage of respondents who were undecided regarding safety of CAMs. In another study, pharmacists' believed that because patients' perceived CAMs as 'safe' and 'natural, ' they were more likely to opt for them rather than conventional medications, which would have been more effective [8]. Overall, the results of attitudes from the current study demonstrate that pharmacists are uncertain of the benefits CAMs have to offer, which can in turn be attributed to a lack of knowledge and understanding.

### Knowledge

During the course of the study, a significant deficit in the tested knowledge of community pharmacists concerning three common CAMs (black cohosh, glucosamine and gingko biloba) was revealed. Firstly, the lack of awareness about hepatotoxicity, an adverse effect of black cohosh, was of particular concern, especially considering the warnings and numerous publications regarding adverse reactions [44-46]. Only 40% of pharmacists were aware of this adverse effect, which was representative of the results in the NPS study. In addition, those who are recommending black cohosh were more likely to know of side effects compared to those who are not recommending the CAM, 42% and 39% respectively. Secondly, glucosamine and its potential to interact with warfarin and cause bleeding has been highlighted within literature [47]. However, only 44% were aware of the interaction and within the NPS study only 38% were aware of the interaction. In addition, 68.4% of respondents in the current study were not sure of potential side effects of glucosamine compared to 21.5% in the NPS study. This is particularly alarming considering the frequency with which glucosamine was reported to have been recommended by pharmacists 100% of the time for either general health, as a preventative or for a specific condition. Further analysis using chi square to analyse differences between groups (never recommending, for general health, as a preventative and for a specific condition) highlighted those who recommended glucosamine for a specific condition were less likely to know of the interaction between glucosamine and warfarin than those who were recommending it as a preventative. Speculating that because glucosamine is generally used in osteoarthritic pain (specific condition) those pharmacists who were aware of its use as a preventative had undertaken further research and were more aware of the drug and specifically the interactions [48]. Previous Australian research confirms that these findings regarding knowledge are consistent, as most pharmacists indicated that their knowledge of CAM therapies was poor [11]. Likewise, a study in the US found that pharmacists were more likely to answer correctly about the uses of CAMs, than adverse effects or cautions, indicating more information is needed on potential risks associated with use [49]. In conclusion, although pharmacists in the current study suggested they were less aware of side effects and interactions in comparison to the NPS respondents, this was not the case. Those in the current study were more knowledgeable in regards to side effects and interactions of CAMs than NPS respondents.

### Information seeking behaviour

Results from the current study indicate that over three quarters of pharmacists needed or sought CAM information several times or more frequently per month. This highlights that pharmacists are seeking information on CAMs and the expectation is that they have adequate resources for their research [33]. In the NPS study, only two thirds of participants sought after information at this frequency. The pharmacists used a wide variety of resources to find information on CAMs, with the internet being the most popular possibly due to the ease of access. It would be interesting to have further investigated the reasons behind the popularity in using the internet for CAM searches. Furthermore, a disparity was evident between usage and perceived usefulness of a list of CAM resources. Those resources which are traditional sources of medicines information for pharmacists rated poorly in usefulness for CAM information, for example dispensing software, MIMS and the Australian Medicines Handbook. The lack of information in these conventional resources about CAMs, in addition to limited training in this area, have both attributed to the poor knowledge of CAMs displayed in the current study. In the NPS study, drug information services (DIS) was the second least likely resource to be used and when it came to usefulness almost 80% reported it as useful. Whereas in the current study, pharmacists used DIS over 50% of the time but it was only found to be useful 32% of the time. Finally, in regards to the type of information pharmacists were seeking, 80% sought information on doses. This particular finding is of interest because doses are always depicted on the primary packaging. The use of qualitative methods such as focus groups or interviews would further explore the reasons for why pharmacists were requesting information on doses, perhaps for off label dosing or because they do not trust the information presented. Although safety of CAMs was the topic pharmacists most frequently reported, 67.5% of those in the current study required information on evidence of effectiveness versus 58.5% in the NPS study.

To date, no published study has addressed the best sources for CAM information for use in the community pharmacy setting. Governing pharmacy bodies can advance this area by increasing awareness of appropriate sources for pharmacy premises. Some pharmacists mentioned the need for a mandatory CAM text. This resonates with a recent survey of pharmacists in Australia in which 27% of pharmacists selling CAM believed they did not have access to CAM information resources such as textbooks or journals at their place of work [13]. Recent research in the US also indicated that many community pharmacists are not satisfied with CAM resources available to them and may not be aware of some of the higher quality resources [21].

Reliable sources of information are necessary for pharmacists' to satisfy the ever increasing demand on CAMs [50]. The PSA supported this notion through their position statement, which recognised the role of pharmacists in providing CAMs information in order to assist patient decisions. The statement explicitly stated that 'pharmacists involved in the supply of such products have the same obligation to provide information and advice, consistent with consumer needs, as they do with registered prescription and proprietary medicines' [51].

### Limitations

As the method involved a self-administered questionnaire, response bias is likely with those displaying greater awareness of CAMs more likely to respond [52,53]. The questionnaire response rate is comparable with other studies examining issues for pharmacists around CAMs in the United States of America and Australia [11,54]. In addition, the current study was found to have a greater response rate than the NPS study. The generalisability of the results is limited as the sample of rural pharmacists was taken from one geographic region which may not be representative of all rural pharmacists. A mixed method study incorporating qualitative methods such as focus groups or interviews would have enabled further data to be gathered from the participants [55,56].

## Conclusions

Pharmacists have varied opinions on the use of CAMs, but the majority studied agree that these medications are now widely accepted by consumers and that pharmacists have a role to play in ensuring that CAMs are used safely and effectively. Many individuals lacked awareness of or access to good quality CAMs information which suggests that it is important to provide pharmacists with information on the best resources and where to access them. In addition, extra opportunities for further education possibly in the form of continuing professional development (CPD) points so that they can better provide advice to consumers on CAM use. The results also indicate the types of information pharmacists desired and how they searched for this information, however further research into identifying how they would like to access this information is required. The findings from this study will be valuable in assisting interested stakeholders with the development of initiatives to address the gaps in attitudes, knowledge and to improve effectiveness of information seeking behaviour. The research, thus far, provides important information regarding rural community pharmacists within the Loddon Mallee region and CAMs. Replication of the study on a larger scale will provide further data relating to rural pharmacists in general. In conclusion, the discussion draws upon findings to demonstrate how CAMs are relevant to the professional practice of pharmacists and highlights the challenges to meeting pharmacists' knowledge requirements and how the CAMs information needs of these health professionals may be supported.

## Competing interests

The authors declare that they have no competing interests.

## Authors' contributions

NB designed and carried out the research project as part of her Pharmacy (Hons) degree. JW, VDS and PW contributed as supervisors of the project. All authors read and approved the final manuscript.

## Pre-publication history

The pre-publication history for this paper can be accessed here:

http://www.biomedcentral.com/1472-6882/11/103/prepub

## Supplementary Material

Additional file 1 **Questionnaire**. Copy of the questionnaire adapted from the NPS for use in this study. Click here for file
