# Supplementary material for: Rural Australian community pharmacists' views on complementary and alternative medicine: a pilot study
Source: BMC Complement Altern Med. 2011 Oct 28;11:103. doi: 10.1186/1472-6882-11-103 (PMC3217877; doi:10.1186/1472-6882-11-103)
Supplement: Additional file 1 — Questionnaire. Copy of the questionnaire adapted from the NPS for use in this study. [file 1472-6882-11-103-S1.PDF]

Appendix 1: Copy of survey

A pilot study of rural community pharmacists views on Complementary and Alternative Medicine.

Thank-you for assisting in this valuable research. If you have any queries, please do not hesitate to contact Nicole Bushett at La Trobe University, Bendigo on 5444-7577

Once you have completed the questionnaire, could you please return it in the enclosed reply paid envelope.

Please tick your response.

Are you currently practicing in community pharmacy?

❑ Yes (Please continue with the questions)
❑ No (No further questions, thank-you. Please return your survey in the Reply-Paid envelope provided).
❑ Both (Please continue with the questions)

Experiences and attitudes on complementary and alternative medicines

1. Please rate the following statements about Complementary and Alternative Medicines (CAMs)

	Strongly disagree	Disagree	Neither agree nor disagree	Agree	Strongly agree
CAMs need more scientific testing before being used in conventional medicine.	❑	❑	❑	❑	❑
CAMs have a more holistic approach to health than conventional medicines.	❑	❑	❑	❑	❑
Most CAMs are safe and have very few side effects.	❑	❑	❑	❑	❑
The results from CAMs are mainly due to a placebo effect.	❑	❑	❑	❑	❑
CAMs can offer patients benefits that conventional medicine cannot.	❑	❑	❑	❑	❑
Pharmacists should regularly ask customers if they are using CAMs	❑	❑	❑	❑	❑
I am confident discussing CAM therapy with customers.	❑	❑	❑	❑	❑

2. In what type of situation have you discussed complementary and alternative medicines with a customer? (Mark as many as apply)

❑ The customer specifically requests a CAM
❑ The customer tells me they are taking a CAM
❑ I initiate discussion about CAMs with a customer
❑ Other (specify) ____________________
❑ CAMs are never discussed with a customer

3. In the last 12 months, how often have you recommended a complementary medicine to a customer?

❑ Frequently
❑ Sometimes
❑ Occasionally
❑ Never

4. When recommending a complementary medicine, do you usually recommend a generic name (e.g. glucosamine), or do you usually recommend a specific brand name to patients?

❑ Brand name
❑ Generic name

5. What influences your recommendation of a specific brand name of a complementary and alternative medicine? (Mark as many as apply)

❑ Brand reputation for quality
❑ Brand familiarity
❑ Lowest price
❑ Clinically-tested standardised formulation
❑ Other (please specify)________________

6. In the last 12 months, have you ever recommended the following complementary medicines for a customer's general health and well being, and/or to prevent future health problems and/or for a specific condition. (Mark as many as apply)

	Never recommend	Yes: for general health	Yes: as a preventative	Yes: for a specific condition
Black cohosh	❑	❑	❑	❑
Calcium	❑	❑	❑	❑
Coenzyme Q10	❑	❑	❑	❑
Echinacea	❑	❑	❑	❑
Fish oil	❑	❑	❑	❑
Gingko biloba	❑	❑	❑	❑
Ginseng	❑	❑	❑	❑
Glucosamine	❑	❑	❑	❑
Natural weight loss products	❑	❑	❑	❑
St. John's wort	❑	❑	❑	❑
Valerian	❑	❑	❑	❑
Multivitamins	❑	❑	❑	❑
Vitamin A	❑	❑	❑	❑
Vitamin C	❑	❑	❑	❑
Vitamin D	❑	❑	❑	❑
Other vitamin/mineral products	❑	❑	❑	❑
Traditional Chinese medicines	❑	❑	❑	❑
Other (please specify) ___________	■	❑	❑	❑

Information about Complementary and Alternative Medicines

7. In the last 12 months, how often have you needed, or looked for, information on CAMs?

- ❑ Every day
- ❑ Several times a week
- ❑ Several times a month
- ❑ Every month
- ❑ Every few months
- ❑ Less often
- ❑ Never

8. In the last 12 months, what information did you want to know about CAMs? (Mark as many as apply)

- ❑ Dose
- ❑ Indication for use
- ❑ Evidence for effectiveness
- ❑ Drug interactions
- ❑ Adverse effects
- ❑ Contraindications
- ❑ Patient information
- ❑ Product selection
- ❑ Use in children
- ❑ Use in pregnancy
- ❑ Supplier/Wholesaler
- ❑ Other (please specify)________________

9. How useful have you found the following sources of information of CAMs? By useful, we mean the source offered the information you needed. (Mark one box in each row)

Information source	Not used	Not at all useful	Somewhat useful	Moderately useful	Mostly useful	Very useful
MIMS/APPguide	❑	❑	❑	❑	❑	❑
Australian Medicines Handbook	❑	❑	❑	❑	❑	❑
APF	❑	❑	❑	❑	❑	❑
Complementary medicine text book	❑	❑	❑	❑	❑	❑
Peer-reviewed medical journal e.g. MJA	❑	❑	❑	❑	❑	❑
Peer-reviewed pharmacy journal	❑	❑	❑	❑	❑	❑
Complementary medicine journal	❑	❑	❑	❑	❑	❑
Professional association journal	❑	❑	❑	❑	❑	❑
Searching the internet	❑	❑	❑	❑	❑	❑
Specific websites	❑	❑	❑	❑	❑	❑
Industry representatives	❑	❑	❑	❑	❑	❑
Industry magazines or materials	❑	❑	❑	❑	❑	❑
Professional seminars or conferences	❑	❑	❑	❑	❑	❑
Dispensing software	❑	❑	❑	❑	❑	❑
Drug information phone services	❑	❑	❑	❑	❑	❑
Colleagues	❑	❑	❑	❑	❑	❑
Other (specify)______________	❑	❑	❑	❑	❑	❑

10. If good quality information was available right now, which of the following products would you like information on? (Mark as many as apply)

- ❑ Black cohosh
- ❑ Coenzyme Q10
- ❑ Echinacea
- ❑ Fish oil
- ❑ Gingko biloba
- ❑ Ginseng
- ❑ Glucosamine
- ❑ Homeopathic medicines
- ❑ St.John's wort
- ❑ Traditional Chinese medicines
- ❑ Valerian
- ❑ Vitamin A
- ❑ Vitamin D
- ❑ Others (specify)__________________

Awareness and knowledge of complementary and alternative medicine

The next three questions are about three commonly used complementary medicines – black cohosh, glucosamine and gingko biloba. **It is important that you answer the questions at face value and not look for the answers. We simply want to know what pharmacists currently know and do not know about these medicines.**

11. Please rate your current awareness of these three CAMs. (One mark per column)

	Ginkgo biloba	Black cohosh	Glucosamine
I have never heard of it	❑	❑	❑
I have heard of it but don't remember any details	❑	❑	❑
I know a little about its uses, side effects and drug interactions	❑	❑	❑
I have a good working knowledge of its uses, side effects and drug interactions	❑	❑	❑

12. Which of the following are potential side effects of these CAMs? (Mark as many as apply)

	Ginkgo biloba	Black cohosh	Glucosamine
Hypertension	❑	❑	❑
Insomnia	❑	❑	❑
Hypertension	❑	❑	❑
Headaches/dizziness	❑	❑	❑
Bleeding disorders	❑	❑	❑
Depression	❑	❑	❑
Osteoporosis	❑	❑	❑
Weight loss/weight gain	❑	❑	❑
Gastrointestinal disturbance	❑	❑	❑
Liver problems	❑	❑	❑
Muscle pains	❑	❑	❑
Seizures	❑	❑	❑
Other (specify) ____________________	❑	❑	❑
No potential side effects	❑	❑	❑
Not sure	❑	❑	❑

13. With which of the following medications do the three complementary medicines potentially interact? (Mark as many as apply)

	Ginkgo biloba	Black cohosh	Glucosamine
Paracetamol	❑	❑	❑
Fluvoxamine	❑	❑	❑
Warfarin	❑	❑	❑
Digoxin	❑	❑	❑
Frusemide	❑	❑	❑
Aspirin	❑	❑	❑
Tamoxifen	❑	❑	❑
Oral contraceptives	❑	❑	❑
Ibuprofen	❑	❑	❑
No potential interactions	❑	❑	❑
Not sure	❑	❑	❑

General Information

This section asks about your background so we can be sure we have a good cross-section of pharmacists in the survey.

14. In the last 12 months, how often have you *personally* used a CAM?

- ❑ Frequently
- ❑ Sometimes
- ❑ Occasionally
- ❑ Never

15. What is your gender?

❑ Male ❑ Female

16. In what year were you born?

[| | |]

17. Did you complete your primary pharmacy degree in Australia?

- ❑ Yes
- ❑ No

18. Have you undertaken any postgraduate CAM qualifications (e.g. Graduate Diploma in Herbal Medicines)?

- ❑ Yes (specify) ___________________
- ❑ No

19. For how many years have you worked as a registered pharmacist?

[|]

20. In which of the following pharmacy field(s) do you work? *Please mark all that apply and indicate hours as appropriate.*

- ❑ Community/ retail pharmacy [|] Hrs/week
- ❑ Hospital/ clinical pharmacy [|] Hrs/week
- ❑ Consultant pharmacy services /Accredited pharmacist [|] Hrs/week

21. What is the postcode of your main place of work?

[| | |]

22. Do you work in a pharmacy that specialises in compounding?

- ❑ Yes (specify specialities)_________________
- ❑ No

23. Do you offer any of the following CAMs in the pharmacy where you work? (Mark as many as possible)

- ❑ Traditional Chinese medicine
- ❑ Naturopathy/ western herbalism
- ❑ Homeopathy
- ❑ Other (specify)________________
- ❑ None

24. Do you consider that you practice Integrative Care (a holistic approach to health care that integrates conventional medical care with complementary therapies?)

- ❑ Yes
- ❑ No

25. In the pharmacy where you work most frequently, what proportion of CAM sales is a pharmacist involved in?

- ❑ All
- ❑ Most
- ❑ About half
- ❑ Some
- ❑ None at all.

Thank-you kindly for completing this survey!

If you have any queries, please do not hesitate to contact me.

Regards,

Nicole Bushett.
